# Mitochondrial genome refinement and comparative phylogenetics of *Parastrongyloides trichosuri* (KNP strain; Nematoda: Strongyloididae) from South Africa

**DOI:** 10.1590/0074-02760250294

**Published:** 2026-04-27

**Authors:** Kwangjae Cho, Minkyung Kim, Jun Won Park, Yang-Kyu Choi, Won Gi Yoo

**Affiliations:** 1 Seoul National University, College of Veterinary Medicine and Research Institute for Veterinary Science, Seoul, Republic of Korea; 2 Konkuk University, College of Veterinary Medicine, Department of Laboratory Animal Medicine, Seoul, Republic of Korea

**Keywords:** Parastrongyloides trichosuri, mitogenome, Strongyloididae, reannotation, reassembly

## Abstract

**BACKGROUND:**

*Parastrongyloides trichosuri* Mackerras, 1959 (Nematoda: Strongyloididae) is a facultatively parasitic nematode infecting the common brushtail possum. A previously reported mitochondrial genome (mitogenome) from a Kruger National Park (KNP) isolate (GenBank: NC_028620) is incomplete, lacking the *nad3* gene and the noncoding region (NCR), limiting its utility for comparative and phylogenetic studies.

**OBJECTIVES:**

This study aimed to reconstruct, annotate, and validate a complete mitogenome of *P. trichosuri* (KNP strain) to enhance genomic accuracy and phylogenetic resolution within Strongyloididae.

**METHODS:**

Whole-genome sequencing data (SRA: ERS056619) were reanalysed. Missing genes, including tRNA-Glu and *atp6*, were manually curated using BLASTn searches against Rfam v15, while *cox3* and *nad3* were confirmed through transmembrane topology analysis.

**FINDINGS:**

The reconstructed mitogenome was 13,809 bp long, comprising 12 protein-coding genes, 22 tRNAs, 2 rRNAs, and a 539-bp tandem-repeat NCR. Gene order and structure were consistent with other Strongyloididae mitogenomes. Phylogenetic analysis supported *P. trichosuri* as a distinct lineage within the family. The annotated sequence has been deposited in the Third Party Annotation database in GenBank (accession No. BK075097).

**MAIN CONCLUSIONS:**

This improved mitogenome fills an existing genomic gaps and provides a reliable reference for future comparative, phylogenetic, and evolutionary studies of Strongyloididae.

The genus *Parastrongyloides* Morgan, 1928 (Nematoda: Strongyloididae) comprises facultatively parasitic nematodes that exhibit both free-living and parasitic generations within their life cycle.^1^ Among them, *Parastrongyloides trichosuri* Mackerras,1959 first described from the brush-tailed possum (*Trichosurus vulpecula*) in Australia, has become a key experimental model for understanding the transition between free-living and parasitic life styles in nematodes. The species is phylogenetically related to *Strongyloides* spp., sharing the ability to produce free-living adults and infective third-stage larvae that develop in the environment and resume parasitic growth upon infection of a suitable mammalian host.^2^ Obtaining and annotating the mitochondrial genome (mitogenome) of *P. trichosuri* is essential for several reasons. First, it will provide baseline genomic information to compare gene content, order, and nucleotide composition with other Rhabditid and Strongyloidid nematodes, shedding light on the evolutionary origin of facultative parasitism.^2^ Second, mitogenomic phylogenies may refine the placement of *Parastrongyloides* relative to *Strongyloides* and allied taxa, complementing nuclear-gene analyses that remain unresolved.^3^ Finally, a well-annotated mitogenome will enrich comparative datasets for elucidating gene-order evolution within Chromadorea and support population-genetic and evolutionary-developmental studies.^4^ Collectively, these perspectives highlight the significance of reconstructing a complete mitogenome of *P. trichosuri*, which will address a major gap in nematode comparative genomics and enhance our understanding of the evolution of parasitism within the Strongyloididae. Although a mitogenome of *P. trichosuri* from Kruger National Park (KNP), South Africa, has previously been deposited in GenBank (accession No. NC_028620; Pt-i), it represents an incomplete sequence lacking both the *nad3* gene and the noncoding region (NCR). In this study, we report a complete mitogenome (Pt-c) of *P. trichosuri* that has been reconstructed and accurately annotated.

## MATERIALS AND METHODS

*Retrieval and preprocessing of genomic data from the NCBI database* - Whole-genome sequence (WGS) data of *P. trichosuri* were retrieved from the ‘50 Helminth Genomes Project’.^2^ The Illumina paired-end reads (2 × 100 bp) were downloaded in the Sequence Read Archive (SRA) under the accession No. ERS056619. Briefly, the raw reads (41.43 Gb each paired-end read sample) were generated using Illumina HiSeq 2000. A total of 38.14 Gb of clean data per paired-end read sample was obtained after adapter removal and quality trimming (Phred score cutoff of 33) using Trimmomatic v0.39.^5^

*Reads filtering and de novo assembly* - The cleaned data were used to reconstruct the complete mitogenome assembly of *P. trichosuri*. *De novo* assembly was performed with GetOrganelle toolkit v1.7.7.1^6^ which has been reported as one of the best-performing assembly pipelines for mitogenome reconstruction.^7^ The seed/label sequence databases were customised using Nematoda RefSeq mitogenomes (Taxonomy ID: 6231). Pre-filtering was applied using the Nematoda RefSeq sequences to enrich mitochondrial reads. The resulting filtered reads were taxonomically classified with Kraken 2 (July 2025 release)^8^ to evaluate potential host or pathogen contamination. The final assembly was generated using the built-in SPAdes v4.2.0^9^ assembler, and quality validation was conducted by remapping the filtered reads with Bowtie 2 v2.5.4^10^ to ensure the absence of detectable contamination. The assembly graph was further inspected and adjusted using Bandage v0.8.1.^11^

*Functional genome annotation and identification of missing genes* - Mitogenome annotation, including the mitochondrial protein-coding genes (mPCG) and functional RNAs, such as transfer RNA (tRNA) and ribosomal RNA (rRNA), was performed using the MITOS2 pipeline^12^ through the GALAXY server v2.1.9 (https://usegalaxy.org/). The open reading frames (ORFs) of the mPCGs were manually confirmed using the ORF finder (https://www.ncbi.nlm.nih.gov/orffinder/) with the invertebrate mitochondrial genetic code and were identified by comparison with the reported mitogenomes of the family Strongyloididae. tRNA-Glu (*trnE*) and *apt6* not detected by MITOS2 were recovered through BLASTn searches against Rfam v15 containing Strongyloididae sequences.^13^ For *cox3* and *nad3*, both ORF and distinctive transmembrane (TM) domains were identified using TMHMM v2.0^14^ and DeepTMHMM v1.0 (https://services.healthtech.dtu.dk/services/DeepTMHMM-1.0/). Repeat units (RUs) were predicted using Tandem Repeats Finder v4.09.1^15^ with default parameters. All gene organisation features were visualised using Proksee v6.0.2.^16^ The Dynamic Genomic Alignment server (DiGAlign) v2.0^17^ was employed with default parameters to compare synteny and perform alignment of genomic elements via BLASTn.

*Mitochondrial phylogenomics* - A total of 12 mPCGs, as well as all individual genes, were retrieved for nine Strongyloididae taxa (including *P. trichosuri*) from NCBI GenBank [Supplementary data (Table)]. Amino acid sequences were obtained in FASTA format and concatenated for the mPCG dataset. Each dataset including concatenation of 12 mPCGs (con-mPCGs) and all individual genes was aligned at the amino acid level using MAFFT v7.505^18^ with default parameters. Ambiguously aligned or poorly conserved regions were manually inspected and removed in MEGA11^19^ prior to phylogenetic analysis. Phylogenetic trees were inferred under the maximum likelihood framework in MEGA11. For each dataset, the Le and Gascuel substitution model with a discrete Gamma distribution to account for among‐site rate variation (five categories) was applied. The shape parameter (α) was estimated from the data. Initial trees for the heuristic search were generated by applying the Neighbor-Joining and BioNJ algorithms to a matrix of pairwise distances estimated using the JTT model, and the topology with the highest log likelihood was selected. Branch support was assessed by bootstrap analysis with 1,000 pseudoreplicates. Two *Schistosoma* species were used as outgroups, specifically *Schistosoma mansoni* and *S. japonicum*, as suggested by Viney [Supplementary data (Table)].^20^

## RESULTS

**Fig. 1:**
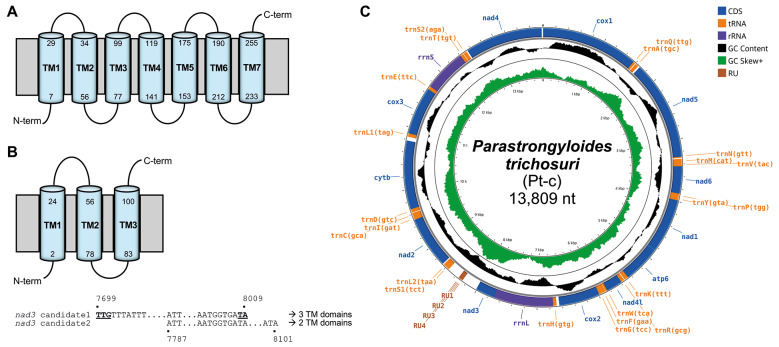
complete mitogenome (Pt-c, BK075097) of *Parastrongyloides trichosuri* showing annotated genomic features. Predicted transmembrane (TM) topologies of *cox3* (A) and *nad3* (B), respectively, showing complete agreement between TMHMM v2.0 and DeepTMHMM v1.0 in the number and position of predicted TM domains. The final *nad3* sequence was determined based on pairwise alignment of two candidates and the consistency of their predicted TM domain counts. Start and stop codons are underlined. (C) Final circular map of the mitogenome.

**Fig. 2:**
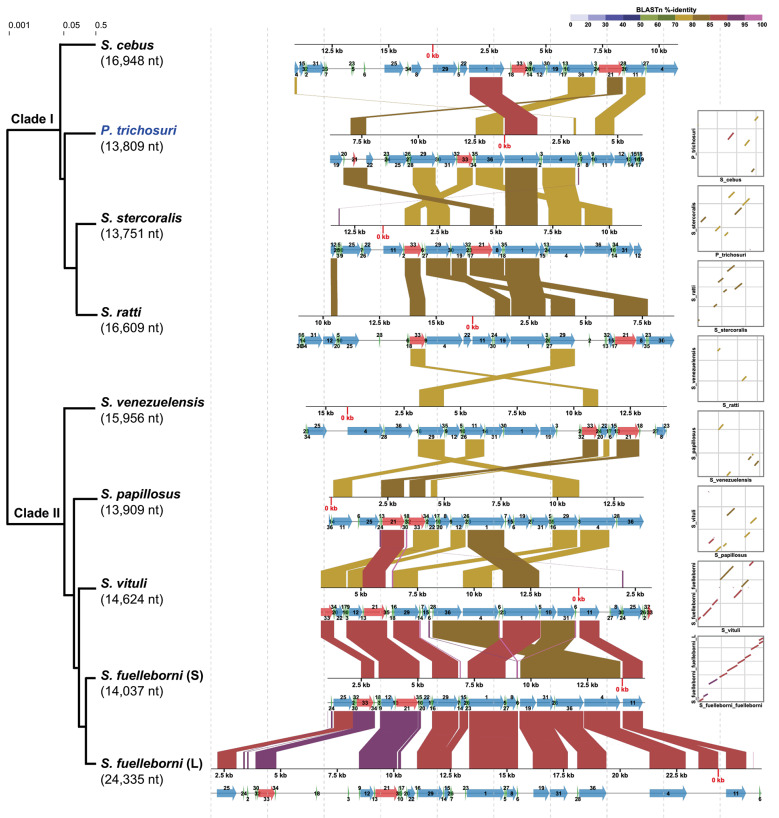
micro-synteny and phylomitogenomic analyses of *Parastrongyloides trichosuri* and major *Strongyloides* species. Regions in adjacent assemblies are connected with coloured ribbons for similar regions in the alignment with Nucleotide %-identity (BLASTn %-identity) coded as colour from light blue to pink. All identified micro-syntenies were automatically aligned and positioned. Blue and red arrows on the assemblies depict the mPCGs and rRNA genes, respectively, while green arrowheads indicate tRNA genes. The rectangular phylogenetic tree is displayed with branch lengths scaled logarithmically. Each of the 36 genes is numbered, and the corresponding gene list is provided in Table I.

*Completion of P. trichosuri mitogenome assembly and annotation* - Reconstruction of *P. trichosuri* mitogenome from WGS data was performed using GetOrganelle toolkit,^6^ which applies preassembly filtering based on organelle-specific seed sequences. Taxonomic classification of the filtered reads using Kraken 2^8^ revealed that no mammalian host DNA contamination was present. However, bacterial contaminants accounted for 48.40% of the total reads, with the majority belonging to the phylum Pseudomonadota (38.76%) [Supplementary data (Fig. 1)]. After *de novo* assembly with the built-in SPAdes^9^ assembler, and subsequent validation by Bowtie 2^10^ remapping, no detectable bacterial sequences remained in the final assembly. The assembly graph, inspected in Bandage,^11^ revealed a single circular contig, with no ambiguous or unresolved regions detected [Supplementary data (Fig. 2)]. The *trnE* and *atp6* were manually annotated using BLASTn searches against the Strongyloididae sequences. In particular, *cox3* and *nad3* were successfully refined and validated through comparative analysis of TM domain topology, which confirmed that *cox3* possesses seven TM domains and *nad3* three ones, respectively (Fig. 1A-B). The resulting well-annotated circular mitogenome measured 13,809 bp in length (Table I, Fig. 1).

**TABLE I:** Gene content, sequence length, and initiation/stop codons of *Parastrongyloides trichosuri* mitogenomes, incomplete mitogenome (Pt-i, NC_028620) and complete mitogenome (Pt-c, BK075097)

Gene/region		Positions and size (bp)		Initiation and termination codons		Anticodon
		Pt-i^a^	Pt-c	Pt-i	Pt-c	Pt-c
1	*cox1*	5,288-6,827 (1,540)	1-1,560 (1,560)	ATG/TAG	ATT/TAA	
2	tRNA-Ala (A)	6,828-6,882 (55)	1,541-1,595 (55)			TGC
3	tRNA-Gln (Q)	6,896-6,949 (54)	1,609-1,662 (54)			TTG
4	*nad5*	6,950-8,531 (1,582)	1,690-3,285 (1,596)	TTG/TTT	ATT/TAA	
5	tRNA-Asn (N)	8,532-8,590 (59)	3,244-3,303 (60)			GTT
6	tRNA-Met (M)	8,606-8,667 (62)	3,319-3,380 (62)			CAT
7	tRNA-Val (V)	8,668-8,722 (55)	3,380-3,436 (57)			TAC
8	*nad6*	8,723-9,160 (438)	3,415-3,873 (459)	ATT/TAA	ATG/TAA	
9	tRNA-Pro (P)	9,161-9,214 (54)	3,874-3,927 (54)			TGG
10	tRNA-Tyr (Y)	9,215-9,271 (57)	3,928-3,984 (57)			GTA
11	*nad1*	9,275-10,746 (870)	3,982-4,857 (786)	ATT/TAG	TTG/TAG	
12	*atp6*	10,147-10,746 (600)	4,860-5,459 (600)	ATT/TAA	ATT/TAA	
13	tRNA-Lys (K)	10,747-10,809 (63)	5,460-5,522 (63)			TTT
14	tRNA-Arg (R)	10,821-10,875 (55)	5,534-5,588 (55)			GCG
15	*nad4L*	10,876-11,107 (232)	5,562-5,850 (289)	TTG/TTT	ATT/GGT	
16	tRNA-Trp (W)	11,108-11,164 (57)	5,821-5,877 (57)			TCA
17	tRNA-Phe (F)	11,168-11,223 (56)	5,881-5,936 (56)			GAA
18	tRNA-Gly (G)	11,225-11,278 (54)	5,938-5,991 (54)			TCC
19	*cox2*	11,225-11,974 (696)	5,962-6,646 (685)	TTG/TAG	TTG/TAT	
20	tRNA-His (H)	11,977-12,031 (55)	6,690-6,744 (55)			GTG
21	*rrnL*	12,032-12,985 (954)	6,745-7,698 (954)			TCA
22	*nad3*	n.a.	7,699-8,009 (311)		TTG/TA^b^	
23	tRNA-Leu (L2)	29-83 (55)	8,549-8,631 (83)			TAA
24	tRNA-Ser (S1)	84-137 (54)	8,604-8,657 (54)			TTC
25	*nad2*	138-978 (841)	8,676-9,503 (828)	TTG/TTT	ATG/TAG	
26	tRNA-Ile (I)	979-1,040 (62)	9,499-9,560 (62)			GAT
27	tRNA-Cys (C)	1,041-1,097 (57)	9,561-9,617 (57)			GCA
28	tRNA-Asp (D)	1,107-1,163 (57)	9,627-9,683 (57)			GTC
29	*cytb*	1,164-2,264 (1,101)	9,657-10,784 (1,128)	TTG/TAA	TTG/TAA	
30	tRNA-Leu (L1)	2,327-2,381 (58)	10,847-10,901 (55)			AGA
31	*cox3*	2,382-3,147 (766)	10,902-11,669 (768)	TTG/ATT	TTG/TGA	
32	tRNA-Glu (E)	3,148-3,202 (55)	11,668-11,722 (55)			TTC
33	*rrnS*	3,203-3,886 (684)	11,722-12,408 (687)			
34	tRNA-Ser (S2)	3,885-3,936 (52)	12,407-12,458 (52)			AGA
35	tRNA-Thr (T)	3,944-4,001 (58)	12,466-12,523 (58)			TGT
36	*nad4*	4,002-5,264 (1,263)	12,545-13,786 (1,242)	TTG/TAA	ATG/TAA	
-	Non-coding region	12,985-28 (743)	8,010-8,548 (539)			
-	Repeat unit	n.a.	8,269-8,343 (75)			

a: the partial mitogenome (Pt-i) starts at 5,288 bp while the complete mitogenome (Pt-c) begins at 1 bp; b: non-canonical codons; n.a.: not available.

**Fig. 3:**
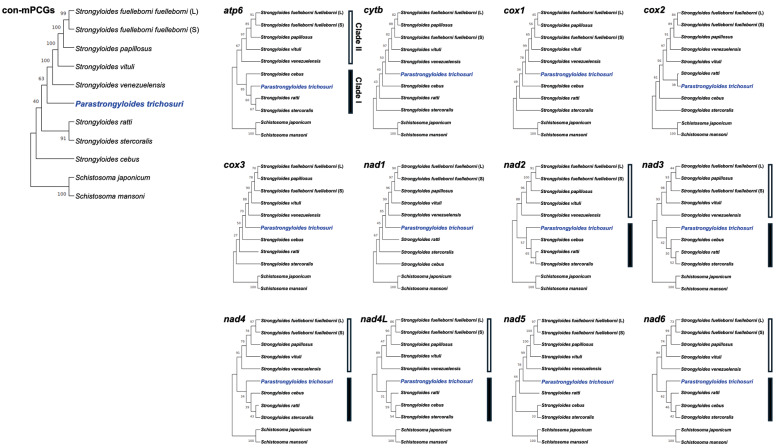
phylogenetic trees showing the position of *Parastrongyloides trichosuri* (highlighted in bold blue). The trees are based on amino acid sequences derived from individual genes and from the concatenation of 12 mitochondrial protein-coding genes (con-mPCGs). *Schistosoma mansoni* and *S. japonicum* were used as outgroups. Nodal support values, estimated from 1,000 bootstrap pseudoreplicates, are indicated at each node. GenBank accession numbers are provided in Supplementary data (Table).

*Structural and compositional features of the complete mitochondrial genome* - Complete and incomplete mitogenomes of *P. trichosuri* were displayed in circular maps showing 36 genes and 35 genes, respectively [Fig. 1C, Supplementary data (Fig. 3)]. The complete mitogenome (Pt-c) of *P. trichosuri* includes 12 mPCGs (*nad1*-*6*, *nad4L*, *cox1*-*3*, *cytb*, *atp6*), 2 rRNA genes (*rrnL*, *rrnS*), and 22 tRNA genes, which is consistent with the mitogenomes of the other *Strongyloides* species (Table I). The total length of the mPCGs was 10,252 bp, which is a proportion of 74.25% of the entire complete mitogenome, compared to 11,567 bp (84.62%) of the incomplete mitogenome (Pt-i). *nad5* (1,596 bp) was the longest while *nad4L* (289 bp) was the shortest of mPCGs. TTG and TAA were the most prevalent codons for initiation and termination, respectively. The whole length of tRNA genes was 1,272 bp, with the length ranging from 52 nucleotides (*trnS2*) to 83 nucleotides (*trnL2*) in the complete mitogenome (Pt-c). Compared with the incomplete mitogenome (Pt-i), the complete mitogenome (Pt-c) revealed the precise annotation of *cox3* and the presence of a 539-bp NCR containing a 75-bp segment composed of three 20-bp RUs and an additional 15-bp short repeat sequence [Table I, Supplementary data (Fig. 4)].

*Comparative mitogenomic analysis of P. trichosuri and other Strongyloides species* - The genetic differences of 12 mPCGs of the mitogenomes of representative species in the family Strongyloididae were compared at the nucleotide level (Table II). The sequence differences between the complete mitogenome (Pt-c) of *P. trichosuri* and the other eight *Strongyloides* species ranged from 27.31% (*S. ratti*) to 43.87% (*S. papillosus*). Interestingly, *S. papillosus* exhibited the greatest divergence across all individual genes. Large differences in sequences were detected in *nad6* (63.32%), *atp6* (63.23%), *nad4L* (57.75%), *nad3* (53.94%), and *nad2* (53.12%).

**TABLE II:** Pairwise distances rate (%) estimation of both individual and concatenated mPCGs and MRGs between *Parastrongyloides trichosuri* and members of the family Strongyloididae

Species/strains^a^	Protein-coding genes (mPCGs)													Mitoribosomal genes (MRGs)		
	atp6	cox1	cox2	cox3	cytb	nad1	nad2	nad3	nad4L	nad4	nad5	nad6	Con-mPCGs	rrnL	rrnS	Con-MRGs
*Strongyloides ratti*	34.35	22.98	**19.93**	27.36	**25.55**	27.36	**34.25**	**27.18**	**25.63**	32.69	**24.87**	**33.31**	**27.31**	22.75	**20.65**	**21.85**
*Strongyloides stercoralis*	**30.03**	20.66	24.16	26.23	27.80	**26.09**	35.83	29.38	35.39	**31.16**	28.97	36.10	27.99	**21.77**	26.05	23.51
*Strongyloides cebus*	44.45	**18.96**	24.67	29.73	28.03	28.41	46.95	33.64	42.32	32.27	30.46	41.00	30.56	24.40	30.18	26.71
*Strongyloides vituli*	52.38	19.03	25.59	28.70	30.31	27.67	38.06	32.21	41.11	31.25	32.71	48.27	30.96	35.19	34.60	34.93
*Strongyloides fuelleborni fuelleborni* (S)	47.18	20.77	26.81	**24.23**	31.25	31.93	41.12	37.04	40.00	32.03	34.57	52.95	32.10	35.55	36.91	36.08
*Strongyloides venezuelensis*	46.93	22.86	33.36	28.78	30.11	27.62	41.71	34.08	35.73	34.47	30.93	48.69	32.18	30.30	35.58	32.39
*Strongyloides fuelleborni fuelleborni* (L)	55.88	23.85	29.79	31.20	35.90	34.47	47.20	42.26	51.90	35.08	40.62	58.91	36.89	36.55	38.51	37.35
*Strongyloides papillosus*	63.23	27.61	35.49	37.21	45.62	39.13	53.12	53.94	57.75	49.55	46.80	63.32	43.87	38.60	45.54	41.37

a: *Parastrongyloides trichosuri* (Pt-c, BK075097) served as the reference for pairwise distance rate calculations. The lowest value for each gene in the pairwise distance matrix was indicated in bold. mPCG, mitochondrial protein-coding gene; MRG: mitoribosomal gene; con-mPCGs: concatenation of 12 mPCGs; con-MRGs: concatenation of 2 MRGs.

Mitogenome-based phylogenetic analysis of *P. trichosuri* and eight *Strongyloides* species revealed two distinct clades, in which *P. trichosuri* formed clade I together with *S. cebus*, *S. stercoralis* and *S. ratti* (Fig. 2). All mitogenomes were compared by automatic repositioning based on gene synteny similarities. Among them, the *P. trichosuri* mitogenome showed extensive rearrangements, consistent with the patterns observed in other *Strongyloides* species. Within clade I, the *cox1* gene was highly conserved, whereas *cytb*, *nad1*, *nad4*, *nad5*, two rRNA genes, and *trnH* exhibited nucleotide identities ranging from 70% to 85%.

*Phylomitogenic analysis* - Phylogenetic relationships are well resolved with high nodal support (Fig. 3). *P. trichosuri* showed a close relationship with three *Strongyloides* speces, such as *S. ratti*, *S. cebus*, and *S. venezuelensis*. Phylogenetic topologies inferred from *apt6*, *nad2*-*4*, *nad4L*, and *nad6* were largely congruent, consistently by revealing two distinct clades. These gene-based phylogenies were consistent with the mitogenome-based phylogenetic tree. Interestingly, the *cox2*-based phylogeny showed *P. trichosuri* forming a distinct group with *S. ratti*, which corresponded to the lowest nucleotide divergence (19.93%) among all mPCGs (Table II).

## DISCUSSION

Mitogenome is informative and robust molecular markers for exploring helminth systematics, population structure, and evolutionary biology.^21^ However, helminth mitogenomes, particularly those of cestodes, exhibit substantial variability in assembly completeness including truncated protein-coding regions or unresolved NCR, and annotation accuracy including missing tRNA genes.^22^ ^,^ ^23^ Similarly, several *Strongyloides* mitogenomes deposited in the NCBI Reference Sequence Database (RefSeq) remain incomplete, such as *S. papillosus* (NC_028622) missing *nad4*, *S. venezuelensis* (NC_028229) and *S. ratti* (NC_028623) missing *trnV*, *S. stercoralis* (NC_028624) missing *trnD*, and *S. cebus* (NC_066659) showing a duplicated *trnN* [Supplementary data (Table)], lacking circularisation or full gene annotation, yet they continue to be applied for numerous researchers without further verification. Therefore, such inappropriate use can lead to systematic bias in mitogenome-based phylogenetic reconstruction and functional interpretation, which is a matter of concern.

*Parastrongyloides trichosuri* mitogenome (LC050209) was originally deposited in the NCBI GenBank in 2016 and was designated as a RefSeq record (NC_028620) in 2023. Unfortunately, *P. trichosuri* mitogenome is incomplete, missing *nad3*, and has been misused as a complete mitogenome in several studies, including tRNA annotation,^24^ and mPCGs-based phylogenomic inference.^4^ ^,^ ^25^ ^,^ ^26^ Remarkably, Ko et al. noted that even the information providing regarding the source species and collection location was unreliable.^24^ Taken together, these observations highlight the urgent need for stricter curation standards and critical assessment of public mitogenome data prior to reuse, underscoring the importance of the complete mitogenome and full annotation of *P. trichosuri* as a reliable reference for future mitogenomic studies.

In mitogenome analysis pipeline, while sample and sequencing quality are undoubtedly important, the choice of a specialised assembler and annotator is even more crucial. Among several assemblers, GetOrganelle toolkit^6^ was employed as the best-performing assembler, as suggested by the benchmarking assembly studies.^7^ To enhance assembly accuracy, a preprocessing step was applied to filter reads using parasite-specific mitochondrial sequences. Notably, in cestodes, the inclusion or omission of this step often determined the success or failure of the assembly.^22^ In addition, this preprocessing step inherently helped to minimise host or pathogen contamination, thereby improving the overall assembly quality.

Within *Parastrongyloides* species, *P. trichosuri* exhibits distinct morphological characteristics, with spicules measuring 80-86 μm in length and having blunt, square-cut ends.^3^ Smales et al.^3^ also reported that *P. trichosuri* is most simlar to *S. stercoralis* (87.6%), followed by *S. ratti* (85.7%) and *S. procyonis* (85.7%) based on partial *cox1* (404 bp) sequence similarity. However, when the phylogeny of nine species was reconstructed using full-length *cox1* sequences, distinct clades were not clearly resolved, and *P. trichosuri* appeared to be more closely related to either *S. cebus* or *S. venezuelensis*, rather than to *S. stercoralis* within the Strongylidae, as shown in Fig. 3. This result highlights the limitations of using partial mitochondrial markers for species-level inference in nematodes.

Comprehensive phylogenomic approaches employing full-length mitogenomes or con-mPCGs may provide more robust evolutionary resolution for *Parastrongyloides* and related taxa. Hunt et al. reconstructed the con-mPCGs-based phylogeny and found that *S. ratti* and *S. stercoralis*, as well as *S. papillosus* and *S. venezuelensis*, each formed distinct clades, with *P. trichosuri* being more closely related to the former clade.^2^ Our mPCGs-based phylogenetic analysis showed a similar pattern.

Our phylomitogenomic analysis clearly distinguished two major clades, clade I (*P. trichosuri*, *S. cebus*, *S. stercoralis*, and *S. ratti*) and clade II (*S. venezuelensis*, *S. papillosus*, *S. vituli*, and *S. fuelleborni fuelleborni*). This pattern was consistent with the individual gene-based phylogenies derived from *atp6*, *nad2*-*4*, *nad4L*, and *nad6*, although the closest relative of *P. trichosuri* occasionally varied between *S. cebus* and *S. ratti*. The pattern of mitochondrial gene rearrangements was broadly congruent with the phylomitogenomic topology. Species within clade I displayed derived rearrangements, particularly involving *cox1* and *rrnL*, whereas those in clade II revealed rearrangements including *nad3*, *cytb*, *rrnS* and *rrnL*. Among these genes, *cytb* and *rrnS* exhibited extensive rearrangements spanning both clade I and clade II, whereas *cox1*, the most typical phylogenetic marker, was rearranged within each clade. Rather than *S. cebus*, *P. trichosuri* appears to have played a more pivotal role in the evolutionary history of the family Strongyloididae. These lineage-specific rearrangements offer key insights, suggesting that the evolution of mitochondrial gene order has been a major driver of diversification within this family.

*In conclusion* - We present an improved and complete mitogenome of *P. trichosuri*, featuring fully resolved gene annotations and a 539-bp NCR. This represents a notable improvement over the previously deposited RefSeq record (NC_028620), which lacks both the *nad3* gene and the NCR. Our findings underscore the importance of employing a parasite-specific mitogenome assembly pipeline, which facilitates the reconstruction of high-quality mitogenomes from WGS datasets. The newly assembled *P. trichosuri* mitogenome provides a reliable and comprehensive resource for future comparative mitogenomic and phylomitogenomic investigations within the Strongyloididae and other related nematode lineages.

## SUPPLEMENTARY MATERIALS

Supplementary material

## Data Availability

The reassembled and reannotated complete mitogenome sequence data of *Parastrongyloides trichosuri* are available in the Third Party Assembly/Annotation Section of the DDBJ/ENA/GenBank databases under the accession number TPA: BK075097.
