# Supplementary material for: Mitochondrial genome refinement and comparative phylogenetics of Parastrongyloides trichosuri (KNP strain; Nematoda: Strongyloididae) from South Africa
Source: Mem Inst Oswaldo Cruz. 2026 Apr 27;121:e250294. doi: 10.1590/0074-02760250294 (PMC13123268; doi:10.1590/0074-02760250294)
Supplement: Supplementary material [file 1678-8060-mioc-121-e250294-s1.pdf]

TABLE

Mitochondrial genome information and definitive hosts of *Parastrongyloides trichosuri*, representative Strongyloididae species and outgroup used in this study

Species/strain	Accession No.	Length (nt)	Annotation	Definitive host[a]	Publication
Strongyloides fuelleborni fuelleborni (L)	PV564809	24,335	36 genes	Non-human primates, human	n.a.
Strongyloides fuelleborni fuelleborni (S)	OL672152	14,037	36 genes	Non-human primates, human	n.a.
Strongyloides papillosus	NC_028622	13,909	*nad4* missing	Ruminants	n.a.
Strongyloides vituli	NC_066507	14,624	36 genes	Cattle	n.a.
Strongyloides venezuelensis	NC_028229	15,956	*trnV* missing	Rodents	n.a.
Parastrongyloides trichosuri	BK075097	13,809	36 genes	Marsupials	This study
Strongyloides ratti	NC_028623	16,609	*trnV* missing	Rat	n.a.
Strongyloides stercoralis	NC_028624	13,751	*trnD* missing	Human, dogs	n.a.
Strongyloides cebus	NC_066659	16,948	*trnN* duplicate	Non-human primates	n.a.
Schistosoma mansoni	NC_002545	14,415	-	Human, other mammalian hosts	(1)
Schistosoma japonicum	KU196417	14,088	-	Human, other mammalian hosts	(2)

a: definitive host information was obtained from.[3] n.a.: not available.

Percent	Clade Reads	Taxon Reads	Rank Code	Taxonomy ID	Scientific Name
50.09	64777263	64777263	U	0	unclassified
**49.91**	**64556180**	**42327**	**R**	**1**	**root**
49.82	64428942	464965	R1	131567	cellular organisms
**48.40**	**62595382**	**884041**	**R2**	**2**	**Bacteria**
43.71	56533339	220035	K	3379134	Pseudomonadati
38.76	50132350	775938	P	1224	Pseudomonadota
0.00	7	0	R3	68525	delta/epsilon subdivisions
0.00	7	0	C	28221	Deltaproteobacteria
0.00	7	0	O	1779134	Bradymonadales
0.00	7	0	F	1779135	Bradymonadaceae
0.00	6	0	G	1779136	Bradymonas
0.00	6	6	S	1548548	Bradymonas sediminis
0.00	1	0	G	2766927	Persicimonas
0.00	1	1	S	2292766	Persicimonas caeni
0.00	1	0	R3	48479	environmental samples
0.00	1	1	S	77133	uncultured bacterium
0.00	1	0	R3	49928	unclassified Bacteria
0.00	1	1	S	2355	unidentified bacterial endosymbiont
**1.06**	**1366052**	**61624**	**R2**	**2759**	**Eukaryota**
0.99	1282505	18285	R3	33154	Opisthokonta
0.94	1214047	0	K	33208	Metazoa
0.94	1214047	0	K1	6072	Eumetazoa
0.94	1214047	0	K2	33213	Bilateria
0.94	1214047	0	K3	33511	Deuterostomia
0.94	1214047	0	P	7711	Chordata
0.94	1214047	0	P1	89593	Craniata
0.94	1214047	0	P2	7742	Vertebrata
0.94	1214047	0	P3	7776	Gnathostomata
0.94	1214047	0	P4	117570	Teleostomi
0.94	1214047	0	P5	117571	Euteleostomi
0.94	1214047	0	P6	8287	Sarcopterygii
0.94	1214047	0	P7	1338369	Dipnotetrapodomorpha
0.94	1214047	0	P8	32523	Tetrapoda
0.94	1214047	0	P9	32524	Amniota
0.94	1214047	0	C	40674	Mammalia
0.94	1214047	0	C1	32525	Theria
0.94	1214047	0	C2	9347	Eutheria
0.94	1214047	0	C3	1437010	Boreoeutheria
0.94	1214047	0	C4	314146	Euarchontoglires
0.94	1214047	0	O	9443	Primates
0.94	1214047	0	O1	376913	Haplorrhini
0.94	1214047	0	O2	314293	Simiiformes
0.94	1214047	0	O3	9526	Catarrhini
0.94	1214047	0	O4	314295	Hominoidea
0.94	1214047	0	F	9604	Hominidae
0.94	1214047	0	F1	207598	Homininae
0.94	1214047	0	G	9605	Homo
0.94	1214047	1214047	S	9606	Homo sapiens

Fig. 1: taxonomic profiling of filtered reads by Kraken 2 reveals bacterial contamination dominated by *Pseudomonadota*.

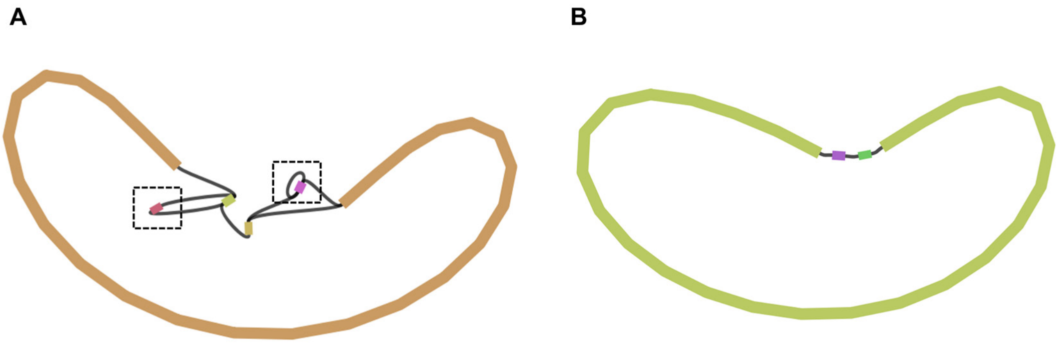

Fig. 2: circular representation of the mitochondrial genome assembly of *Parastrongyloides trichosuri* in this study, visualized using Bandage v0.8.1. (A) The initial assembly failed to form a circular genome due to the presence of two repeated fragments (indicated by the dotted box). (B) A circular mitochondrial genome was successfully reconstructed by manually removing the two repeated fragments.

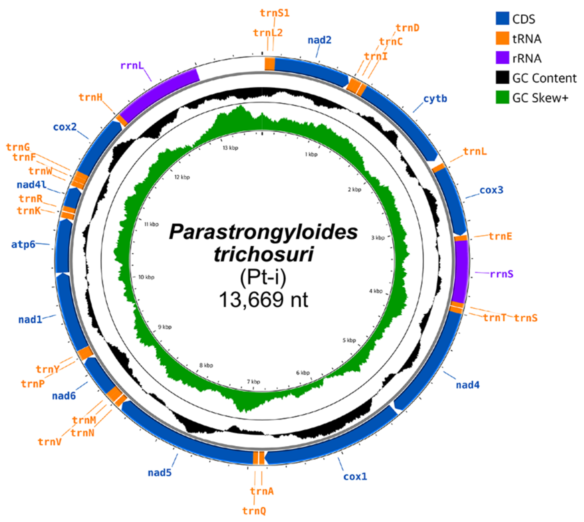

Fig. 3: circular map of the incomplete mitogenome of *Parastrongyloides trichosuri* (Pt-i; GenBank: NC_028620).

```
Indices: 8269-8343  Score: 150
   Period size: 20  Copynumber: 3.8  Consensus size: 20

      8269 TATGTAAAGTTTTATTTTAA
         1 TATGTAAAGTTTTATTTTAA

      8289 TATGTAAAGTTTTATTTTAA
         1 TATGTAAAGTTTTATTTTAA

      8309 TATGTAAAGTTTTATTTTAA
         1 TATGTAAAGTTTTATTTTAA

      8329 TATGTAAAGTTTTAT
         1 TATGTAAAGTTTTAT

Statistics
Matches: 55,  Mismatches: 0, Indels: 0
        1.00            0.00        0.00

Matches are distributed among these distances:
  20   55  1.00

ACGT count: A:0.35, C:0.00, G:0.11, T:0.55
```

Fig. 4: a 75-bp repeat segment identified in the noncoding region. Green, purple, and blue indicate three 20-bp repeat units, and orange shows an additional 15-bp short repeat sequence.

REFERENCES

1. Le TH, Blair D, Agatsuma T, Humair P-F, Campbell NJH, Iwagami M, et al. Phylogenies inferred from mitochondrial gene orders — a cautionary tale from the parasitic flatworms. Mol Biol Evol. 2000; 17(7): 1123-5.
2. Yin M, Zheng HX, Su J, Feng Z, McManus DP, Zhou XN, et al. Co-dispersal of the blood fluke *Schistosoma japonicum* and *Homo sapiens* in the Neolithic Age. Sci Rep. 2015; 5: 18058.
3. Hunt VL, Tsai IJ, Coghlan A, Reid AJ, Holroyd N, Foth BJ, et al. The genomic basis of parasitism in the *Strongyloides* clade of nematodes. Nat Genet. 2016; 48(3): 299-307.
